# Hemophagocytic Lymphohistiocytosis During HIV Infection in Cayenne Hospital 2012–2015: First Think Histoplasmosis

**DOI:** 10.3389/fcimb.2020.574584

**Published:** 2020-09-24

**Authors:** Duc Nguyen, Mathieu Nacher, Loic Epelboin, Alessia Melzani, Magalie Demar, Denis Blanchet, Romain Blaizot, Kinan Drak Alsibai, Philippe Abboud, Félix Djossou, Pierre Couppié, Antoine Adenis

**Affiliations:** ^1^Centre d'Investigation Clinique Antilles Guyane, INSERM 1424, Centre Hospitalier de Cayenne, Cayenne, French Guiana; ^2^Service des Maladies Infectieuses et Tropicales, Centre Hospitalier de Cayenne, Cayenne, French Guiana; ^3^COREVIH Guyane, Centre Hospitalier de Cayenne, Cayenne, French Guiana; ^4^DFR Santé, Université de Guyane, Cayenne, French Guiana; ^5^UMR TBIP, Université de Guyane, Cayenne, French Guiana; ^6^Laboratory, Centre Hospitalier de Cayenne, Cayenne, French Guiana; ^7^Service de Dermatologie-Vénéréologie, Centre Hospitalier de Cayenne, Cayenne, French Guiana; ^8^Service d'Anatomopathologie, Centre Hospitalier de Cayenne, Cayenne, French Guiana

**Keywords:** haemophagocytic lymphohistiocytosis, reactive hemophagocytic syndrome, macrophage activation syndrome, HIV, histoplasmosis, liposomal amphotericin B, French Guiana

## Abstract

**Introduction:** Haemophagocytic Lymphohistiocytosis (HLH), during HIV infection is a rare complication with a poor prognosis. There are few data on HLH within the Amazon region. The objective was to describe epidemiological, clinical and therapeutic features of HIV-related HLH in French Guiana.

**Methods:** A retrospective analysis of adult HIV patients at Cayenne hospital with HLH between 2012 and 2015. A diagnosis of HLH was given if the patient presented at least 3 of 8 criteria of the HLH-2004 classification.

**Results:** Fourteen cases of HLH were tallied during the study period. The mean age was 46 years with a sex ratio of 1.8. The most frequent etiology of HLH was an associated infection (12/14). Confirmed disseminated histoplasmosis, was found in 10 of 14 cases, and it was suspected in 2 other cases. The CD4 count was below 200/mm^3^ in 13/14 cases. An HIV viral load >100,000 copies/ml was observed in 13/14 cases. An early treatment with liposomal amphotericin B was initiated in 12/14 cases. The outcome was favorable in 12/14 of all cases and in 10/12 cases involving histoplasmosis. Case fatality was 2/14 among all cases (14.3%) et 1/10 among confirmed disseminated histoplasmosis with HLH (10%). During the study period 1 in 5 cases of known HIV-associated disseminated histoplasmosis in French Guiana was HLH.

**Conclusion:** Histoplasmosis was the most frequent etiology associated with HLH in HIV-infected patients in French Guiana. The prognosis of HLH remains severe. However, a probabilistic empirical first line treatment with liposomal amphotericin B seemed to have a favorable impact on patient survival.

## Introduction

French Guiana is the French overseas territory where the HIV epidemic is most important (Nacher et al., 2018). The most frequent opportunistic infection in HIV patients is disseminated histoplasmosis, and it has long been the first cause of AIDS-related death (Nacher et al., 2011). Hence the overall incidence of disseminated histoplasmosis in a cohort of HIV-infected persons was 1.5 per 100 person-years, but for persons with CD4 counts <50 per mm^3^ it was >10 per 100 person-years (Nacher et al., 2014). For tuberculosis, the incidence in the HIV cohort was 0.9 per 100-person years. In Latin America, it has been described as a neglected disease (Nacher et al., 2013), and the burden of disseminated histoplasmosis among persons with HIV is estimated to be on par with that of tuberculosis (Nacher et al., 2016; Adenis et al., 2018). The Haemophagocytic Lymphohistiocytosis (HLH) is a rare, but often fatal, syndrome of immune hyperactivation corresponding to a cytokine storm. The HLH-2004 definition criteria require either a molecular diagnosis or five of the eight following criteria: fever, splenomegaly, cytopenias affecting at least two of three lineages, hyperferritinemia, hypertriglyceridemia and/or hypofibrinogenemia, low or absent natural killer (NK)-cell activity, elevated soluble CD25 and confirmatory evidence of hemophagocytosis in the bone marrow, the spleen or the lymph nodes (Henter et al., 2007). Genetic mutations can cause HLH, usually in children. Trigger factors such as infection, immunodeficiency, neoplasm, autoimmunity commonly causes HLH in adults (Hayden et al., 2016). HLH has been previously reported among HIV patients with and without disseminated infections. HLH is notably a known complication of acute disseminated histoplasmosis in both HIV+ and HIV- patients (Subedee and Van Sickels, 2015; Townsend et al., 2015; Jabr et al., 2019). Besides histoplamosis, the main etiology for HLH in AIDS patients are Kaposi sarcoma or lymphoma (B cell, T cell, or primary effusion lymphoma), but also toxoplasmosis, CMV disease, mycobacterial infections, and other opportunistic infections, such as candidiasis and pneumocystosis (Bhatia et al., 2003). HLH may also occur during primary HIV infection or Immune Reconstitution Inflammatory Syndrome (IRIS) (Sun et al., 2004; Breton et al., 2006; Melzani et al., 2020). Treatment mostly relies on treatment of histoplasmosis and HIV, corticosteroids, certain chemotherapeutic agents (etoposide), and interleukin-1 receptor antagonists and immune globulins (Ocon et al., 2017). HLH during HIV infection remains a rare event, which has been rarely described, especially in our Amazonian context. Our initial hypothesis was that disseminated histoplasmosis was the main cause of HLH in HIV-infected persons and that antifungals targeting this pathogen would have a beneficial role on prognosis. We therefore conducted an observational retrospective study with the objectives of describing the particularities of HLH in HIV-infected adults in French Guiana.

## Methods

### Study Type

The study was observational, monocentric, descriptive, and retrospective between 01/01/2012 and 05/01/2016.

#### Study Site

The study involved the hospital wards caring for HIV-infected patients requiring hospitalization.

#### Study Population

**Figure 1:**
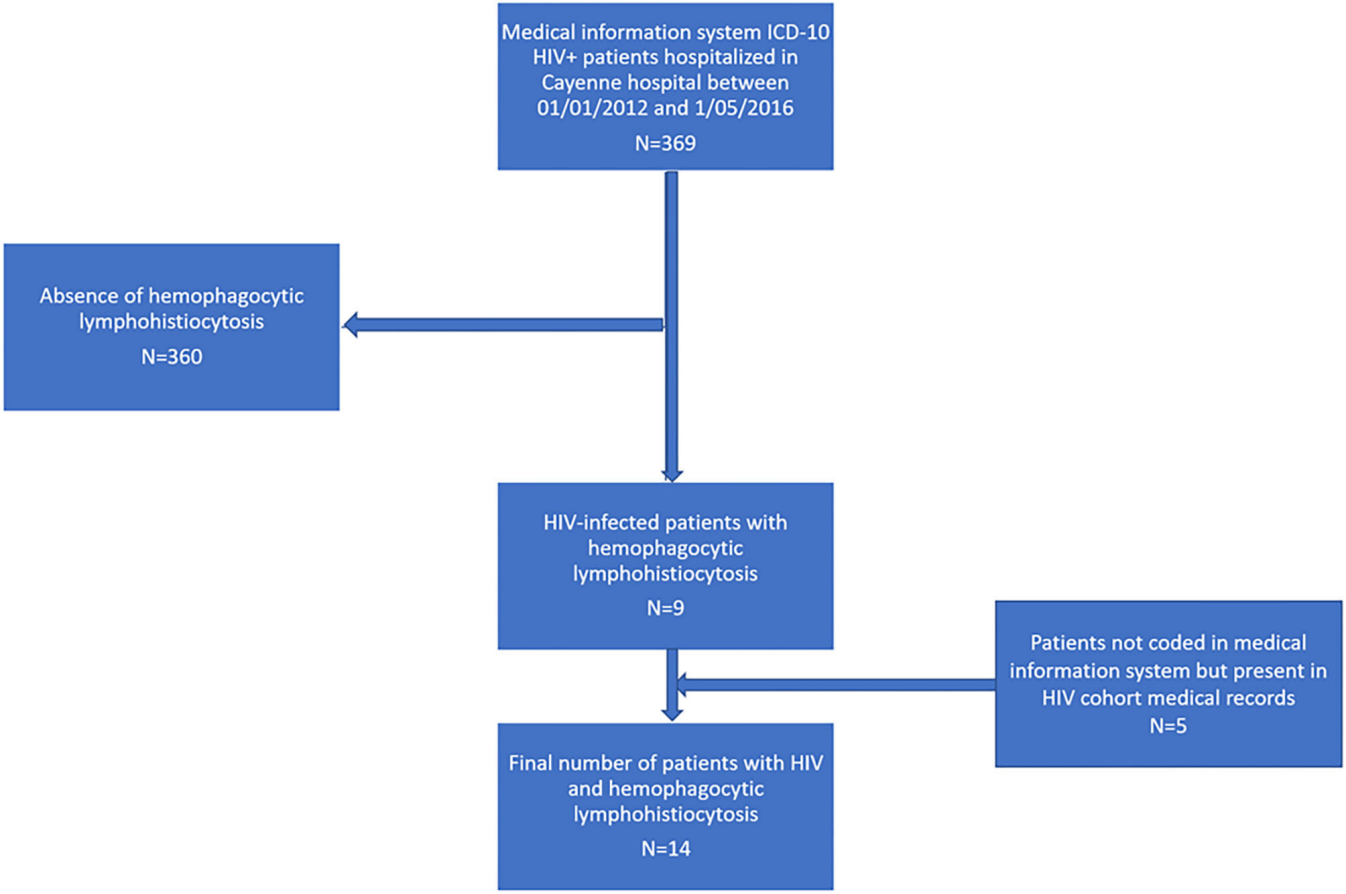
Inclusion diagram of HIV-infected patients with a hemophagocytic lymphohistiocytosis in Cayenne Hospital between 2012 and 2015.

The source population consisted of patients hospitalized in Cayenne hospital between 01/01/2012 and 05/01/2016 and with HIV-specific ICD 10 principal or associated diagnostic codes in the hospital information database. Another source was HIV infected patients included in the HIV computerized medical records eNADIS® (Figure 1). The target population consisted of patients having presented a HLH during their hospital stay.

#### Inclusion Criteria

- Person aged ≥ 18 years
- Confirmed HIV Infection (2 ELISA tests and 1 Western-Blot confirmation).
- ICD 10 Codes CIM-10 as principal diagnosis or associated diagnosis: hemophagocytic syndrome (D761, D762, D763) and HIV infection (B20, B21, B22, B23, B24).
- Diagnosis of the HLH in the eNADIS computerized medical records.

#### Non-inclusion Criteria

- Less than 3 of 8 diagnostic criteria HLH-2004 (Henter et al., 2007).
- HScore (Fardet et al., 2014) below 169 corresponding to a sensitivity of 93% and a specificity of 86%.

#### Principal Judgement Criteria

The main judgement criterion was the presence or absence of a HLH defined according to the HLH-2004 diagnostic criteria (Henter et al., 2007).

A diagnosis of HLH was given if the patient presented at least 3 of 8 criteria of the HLH-2004 classification (Henter et al., 2007). This definition was chosen in order to be more sensitive, and thus to avoid missing cases. A probability HScore was given for each patient as defined by Fardet et al. (2014) in order to refine the specificity of the diagnosis.

#### Data Collection

The identification of target patients required queries in the hospital medical information system Programme de Médicalisation des Systèmes d'Information (PMSI, which includes the main diagnoses and is used to calibrate hospital funding) and eNADIS® a specialized patient record system for HIV patients, which includes detailed follow up data. The modalities of the queries were guided by the ICD-10 codes for HLH as principal diagnosis which allowed obtaining patient identities and dates of hospitalization. Medical records were then reviewed before inclusion in order to extract information. The eNADIS® medical records of the Hospital cohort (>1,200 outpatients, ~90 hospitalizations of HIV patients with opportunistic infections per year; Figure 1) were also searched to identify records with a history of HLH.

For included patients, data were retrospectively collected at Cayenne General Hospital from medical records. Data collection included socio-demographic variables (sex, age, and country of birth), medical data (including general condition, fever, pulmonary signs, digestive signs, lymphadenopathies.) imagery data (chest X ray, CT scan, ultrasound, endoscopy results), biological results (hemoglobin, leukocytes, CRP, LDH, ferritin, albumin etc.), microbiological data (direct examination, culture, pathology, PCR), immunovirological data (CD4 and CD8 counts, viral load before antiretroviral therapy and at the time of HLH) therapeutic data (antifungals, antibiotics, corticosteroids, antiretrovirals.) and the last consultation date after the considered HLH.

In conformity with the diagnostic criteria defining HLH, the highest values during hospitalization of the variables: temperature, aspartate aminotransferase (ASAT), triglycerides and ferritine, and the lowest values of the variables hemoglobin, leukocytes, platelets, fibrinogen were specifically collected at the time of hospitalization for this study.

#### Statistical Analysis

The data from the study were analyzed using Stata 12 (®College Station, Texas, USA) and Microsoft Excel 2013. Descriptive analysis allowed describing sociodemographic, clinical, immunovirological, and therapeutic data. Means and standard deviations, or medians and interquartile ranges, were used depending on the variable distribution.

#### Ethical and Regulatory Aspects

All HIV-infected patients followed at Cayenne Hospital are included in the DAT'AIDS® database. This database allows the clinical care of HIV patients with the eNADIS® computerized medical records. It is part of the French Hospital Database on HIV (FHDH), a national cohort which has led to numerous scientific publications. This database received approval from the Commission Nationale Informatique et Libertés (CNIL) since 27/11/1991. All included patients signed an informed consent form after presentation of the database by the treating physician. The present analysis was hence part of the ongoing approved hospital cohort study and did not require any additional approval.

## Results

This retrospective study allowed identifying 14 HIV-infected patients presenting a confirmed or suspected HLH between the 01/01/2012 and the 05/01/2016. Figure 1 shows the study flowchart. During the time period considered there were 57 cases of disseminated histoplasmosis in Cayenne Hospital, among which 10 (17.5%) were HLH cases.

### Diagnostic Criteria for Patients With HLH

In our series, 3 patients presented only 3 of 8 HLH-2004 criteria and 6 patients presented at least 5 of 8 HLH-2004 criteria. The HScore was >200, corresponding to a probability of 89% to have a HLH in 13/14 patients. Only one patient had an HScore of 183 and 3 of 8 HLH-2004 criteria (Table 1).

**Table 1:** Diagnostic criteria for hemophagocytic lymphohistiocytosis and patient HScore.

**Patient**	**Number of HLH-2004 criteria**	**HScore**	**Probability to have a hemophagocytosis syndrome (%)**
1	3	183	72.23
2	3	237	98.66
3	5	208	92.45
4	5	276	99.87
5	4	279	99.9
6	3	217	95.53
7	4	233	98.23
8	4	233	98.23
9	4	263	99.73
10	4	237	98.63
11	5	202	89.41
12	5	266	99.77
13	6	286	99.93
14	5	256	99.58

### Sociodemographic Characteristics of Patients With HLH

Among the 14 patients, 9 were males (M/F sex ratio = 1.8), the mean age was 46 ± 6 years. Among 14 patients 4 were French born, including French territories of America, and 10 were born in a foreign country. Overall, 9/14 were unemployed and 5/14 had a job; 10/14 cases had health insurance (6 had normal health insurance and 4 had health insurance for precarious populations) and 4/14 had no health insurance.

### Clinical Characteristics of Patients With HLH

In this case series, an advanced HIV stage (stage C3) was observed in most patients (11/14), and all but one patient had a temperature > 38°C (mean = 39.3°C ± 0.8) with varied clinical presentations. Only 3 patients did not have hepatomegaly (5/14) or splenomegaly (7/14) or lymphadenopathy (9/14). Two patients had a complete clinical presentation with all 4 signs (Table 2).

**Table 2:** Clinical criteria of patients with hemophagocytic lymphohistiocytosis.

**Patient**	**Age category (years)**	**CDC (stage)**	**Temperature**	**Adenopathy**	**Hepatomegaly**	**Splenomegaly**	**History of opportunistic infection**	**CD4 count/mm^**3**^**	**Viral load (copies/mm^**3**^)**	**On antiretrovial at the time of HLH**	**Hemoglobin g/DL**	Leukocytes ^*^1,000/mm^**3**^	Platelet count ^*^1,000/mm^**3**^	**Ferritine μ g/ml**	**Triglycerides mmol/L**	**Fibrinogen g/L**	**ASAT (IU/L)**	**Cytological hemophagocytosis**	**Etiology of HLH**	**Diagnosis of histoplasmosis**	**Treatment**	**Outcome**
1	[30–40]	C3	38.4	N	N	N	Y	15	5.9	N	4.7	1.9	8	3.4	2.3	3.8	102	N	DH^*^	Blood + Bone marrow +	LAmB started day 1 +Itraconazole +blood derived products	ICU Died at day 2
2	[50–60]	C3	40	Y	Y	N	Y	4	5.2	N	6.5	2.1	44	9.6	1.9	3.2	96	N	DH^*^+ Salmonella^¶^	Blood + Bone marrow + PCR + Pathology +	LAmB started day 0 +Itraconazole +blood derived products	Discharged at day 24
3	[30–40]	C3	38	N	Y	Y	Y	25	6.4	Y	9.2	4.7	251	1.3	3.7	1.2	148	Y	DH^*^	Blood + Bone marrow + Pathology +	LAmB started day 7 +Itraconazole	Discharged at day 24
4	[40–50]	C3	38.5	Y	Y	Y	Y	14	5.1	N	7.2	13.1	21	23.2	4.1	1.88	1032	N	DH^*^	Blood + Bone marrow + Pathology +	LAmB started day 2 + Steroids +Immunoglobulins	ICU Discharged at day 10
5	[40–50]	C3	40	Y	N	N	N	166	6.8	N	8	1.9	20	7.2	1.8	2.3	352	Y	DH^*^+ CMV	Blood + Bone marrow + Pathology +	LAmB started day 15 +Itraconazole +blood derived products	Discharged at day 80
6	[60–70]	C3	39.4	Y	Y	N	Y	9	5.6	Y	7.9	3.3	80	2.3	1.9	1.6	601	N	DH^*^	Blood + Bone marrow +	LAmB started day 0 +Itraconazole	Discharged at day 14
7	[50–60]	C3	39	Y	N	N	Y	22	5.3	N	7.9	4.6	5	100	1.6	3.4	2409	Y	E Coli^¶^+ Toxic hepatitis	Negative	Steroids +blood derived products	Discharged at day 73
8	[40–50]	C3	38.7	N	N	N	Y	20	5.4	N	8.2	1.9	79	6.5	1.6	3.8	39	Y	DH^*^^†^	Negative	LAmB +Itraconazole	Discharged at day 24
9	[40–50]	B1	38.5	N	N	N	N	837	5.1	Y	6.8	3.4	42	19	2.5	1.6	1586	Y	DRESS syndrome^‡^	Negative		ICU Discharged at day 34
10	[40–50]	C3	37.3	Y	N	Y	N	25	5.2	N	7.9	2.7	27	73.7	1.6	3.8	152	N	DH^*^	Blood + Bone marrow +	LAmB started day 1 +Itraconazole +blood derived products	ICU Discharged at day 14
11	[40–50]	C3	38.9	Y	N	Y	N	25	6.4	N	8.5	6.5	14	14	1.4	2.8	184	Y	DH^*^+ Dengue/CMV reactivation	Blood + Bone marrow + Pathology +	LAmB started day 3 +Itraconazole +Etoposide +blood derived products	Discharged at day 45
12	[40–50]	B1	39.2	N	N	Y	N	36	1.4	Y	9.9	2.2	81	18.1	4.9	2.5	190	Y	DH^*^^†^ E Coli^¶^	Negative	LAmB started day 11 +Itraconazole +blood derived products	ICU Died at day 30
13	[40–50]	C3	39.2	Y	N	Y	N	25	6.7	N	6.9	0.8	6	38.1	3.9	1.6	128	Y	DH^*^	Blood + Bone marrow +	LAmB started day 3 +Itraconazole + Steroids+blood derived products	Discharged at day 63
14	[50–60]	C3	38.5	Y	Y	Y	Y	25	6.4	N	7.2	0.5	42	28	5.4	2.3	123	N	DH^*^	Blood + Bone marrow +	LAmB started day 6	Discharged at day 54

Y, Yes; N, No;

* DH, Disseminated Histoplasmosis;

† suspected diagnosis of histoplasmosis;

‡ drug reaction with eosinophilia and systemic symptoms (DRESS syndrome) ciprofloxacine and piperacillin;

¶ bacteriemia; Patients 1 and 12 died of multiple organ failure and hemophagocytic syndrome. ^*^Aspartate aminotransferase (ASAT).

*Lamb, Liposomal amphotericin B*.

### Immunovirological Status of Patients With HLH

A history of opportunistic infection was described for 8 patients. The number of patients on antiretroviral treatment at the time of the HLH was 4 out of 14. The CD4 count at the time of HLH diagnosis was below 250/mm^3^ in 13 cases (median 25 per mm^3^ Interquartile range = 16–25 per mm^3^). Only one patient had CD4 counts above 500/mm^3^. The HIV viral load was >100 000 copies/ml in 13 out of 14 cases (median 341,097 copies/mm^3^. Interquartile range = 159 374–2 551 033).

### Biological and Cytological Signs in Patients With HLH

Anemia (with hemoglobin <9 g/dl) was present in 13 patients. Leucopenia (leukocytes <4,000/mm^3^) and low platelets (platelets <100,000/mm^3^) were, respectively, observed in 10 and 13 out of 14 cases (Table 2).

Increased ferritin (ferritin > 500 ng/ml) and elevated ASAT were observed in all patients. The median triglyceride concentration was 3 mmol/L (IQR = 2.12–5.2). The median fibrinogen concentration was 2.9 g/L (IQR = 2.4–4.6 g/L).

The presence of signs of hemophagocytosis were present on the bone marrow examination for 8 patients.

### Causes of HLH in HIV Patients in French Guiana

Among the causes observed in patients with HLH, infectious causes were predominant (12/14) with a predominance of fungal causes, notably disseminated histoplasmosis with 10 confirmed cases and 2 suspicions treated as histoplasmosis (12/14) (Table 2).

Fungal/bacterial coinfections were observed in 2 of 14 cases, and fungal/viral coinfections in 2 of the 14 cases. Two non-infectious causes were observed and were linked to a drug reaction, one of which was a Drug Reaction with Eosinophilia and Systemic Symptoms (DRESS), the other being a toxic hepatitis associated with an *E. Coli* infection.

### Paraclinical Diagnosis of HLH in Reaction to Histoplasmosis

The etiologic diagnosis of HLH consecutive to histoplasmosis was based on positive fungal blood cultures in most cases (10/12) (Table 2). In 9 cases, *Histoplasma capsulatum* was simultaneously found in fungal blood cultures and on bone marrow culture. Histoplasmosis serology was negative in all patients. *Histoplasma* PCR was only found in 1 case. Pathological examination was contributive in 5 of 12 cases. In 2 of 12 cases, *Histoplasma capsulatum* could not be identified in any sample, the etiologic diagnostic resting on a favorable evolution on probabilistic antifungal therapy.

### Treatment and Evolution of Patients With HLH

The etiologic treatment with first line systemic antifungal treatment with liposomal amphotericin B was given in 12 of 14 HLH (Table 2). Relay antifungal treatment with itraconazole was also initiated in 10 of 14 cases. Only 1 patient, who probably had a HLH linked to a DRESS syndrome did not receive systemic antifungals.

Among the 4 patients having received probabilistic antifungal treatment, histoplasmosis was not retained as the etiologic agent of HLH for patient n°7. A drug-related hepatitis was suspected in this patient on antibiotics for a urinary tract infection and negative mycological examinations.

Two patients (n°8 and 12) received presumptive antifungal treatment despite negative samples for histoplasmosis. A favorable evolution without any other treatment than antifungals and without any associated pathogen was considered as plausibly indicating histoplasmosis despite the lack of formal evidence.

Specific treatment for HLH was only given in 4 patients with the use of corticosteroids, immunoglobulins and etoposide. The use of blood products was observed in 9 of 14 cases. The delay for initiating antifungal therapy was short with a median of 3 days (IQR: 1–8 days). Five patients presented a severe form of HLH requiring transfer in intensive care unit (ICU), with a fatal evolution in 2 cases. For patient n°1, death occurred within 24 h of admission in ICU and initiation of liposomal amphotericin B. For patient n°12, death was observed 19 days after the initiation of liposomal amphotericin B and 18 days after admission in ICU. The duration of hospitalization was often long with a median of 27 days (IQR: 16–52 days). Case fatality was 2/14 among all cases (14%) et 1/10 among confirmed disseminated histoplasmosis (DH) with HLH (10%), and 2/12 among confirmed and suspected (17%).

## Discussion

In the present study we described 14 HIV-infected patients having presented HLH in a 4.5 year period in Cayenne hospital during which a total 369 patients with HIV were hospitalized. Most patients had severe immunosuppression (most had CD4 counts <100/mm^3^) in the absence of antiretroviral treatment. The diagnosis of HLH was based on an HScore > 200 with a probability > 89 % in 13/14 patients in this series. The main trigger was an infectious pathogen, *Histoplasma capsulatum*, in HLH. Few concomitant coinfections were associated with HLH. The treatment of the infectious trigger was mostly systemic antifungal therapy targeting *Histoplasma capsulatum*, with a rapid initiation delay (3 days). The observed mortality was 14 % for all HLH (2/14) and 10% (1/10) for HLH linked to confirmed disseminated histoplasmosis.

This study was retrospective based on different databases which rely on clinician subjectivity in coding. Some of the diagnostic criteria are specific and may not have been systematically sought for all patients. In addition, there may have been selection biases in Cayenne hospital. Although this small series does not allow statistical inferences, it allows observing general trends on HLH, in HIV patients.

HLH is a complex pathology with variable organ tropism, accounting for the diversity of clinical and biological signs and the absence of pathognomonic elements. Historically, the diagnosis of HLH rested on HLH-2004 criteria proposed by the Histiocyte Society (Henter et al., 2007). These criteria are mixed with clinical, biological, and cytological criteria, and a total of at least 5 of 8 criteria is required. The absence of weighting of these criteria represents a diagnostic difficulty. Fardet et al. proposed a diagnostic score for HLH (HScore) from a multicentric cohort of 162 patients with HLH (Fardet et al., 2014). In our study, the diagnostic of HLH was given if the patient had at least 3 of 8 HLH-2004 criteria instead of the 5 criteria in the HLH-2004 definition. This less restrictive definition was chosen in order to be more sensitive, and thus to avoid missing cases. For 2 of the 14 cases, an HScore value > 200 reflected the interest of this less stringent definition. The patient with an HScore <200 and 3 of 8 HLH-2004 criteria could presumably not express the other criteria because of a short hospitalization and a rapid death at day 2.

Moreover, the cytological element of hemophagocytosis was absent in 6 cases (42 %) and an HScore showing a probability of HLH >90% was observed in 12 cases (85%). This is consistent with other studies showing that the absence of hemophagocytosis on the bone marrow smear was observed in 30% of patients (Fardet et al., 2014). These results suggest that the cytological criterion lacks sensitivity and that the diagnosis of HLH should not be excluded in the absence of this criterion.

The determination of the activity of NK cells and soluble CD25 were not performed here because these analyses are not part of routine explorations. These 2 criteria are often not taken into account by different authors and are not mandatory to assert the HLH diagnosis. Few articles in the literature mention NK activity or soluble CD25 (Karras, 2009; Kolopp-Sarda and Malcus, 2014; Grom et al., 2016). These 2 markers are also not included in the calculation of the HScore.

The specificity of HLH among HIV-infected patients in French Guiana is that its etiology is mostly infectious and mostly due to the disseminated infection by the fungus *Histoplasma capsulatum*. These results are different from those observed in mainland France but are consistent with previous studies on causes of death by opportunistic agents in French Guiana emphasizing differences in pathogen ecology between Europe and the Amazonian area (Nacher et al., 2011). Our observations also contrast with a retrospective study in São Paulo, Brazil, where the main causes of HLH in HIV patients were disseminated histoplasmosis only represented 8% of the trigger factors, with mycobacteria (34%), cytomegalovirus (14%), and cryptococcus (11%) representing the leading causal pathogens. The bibliographic data are varied on the causes of HLH in HIV patients (Koduri et al., 1995; Fiszbin et al., 1998; Fardet et al., 2003; Sproat et al., 2003; Concetta et al., 2004; Sun et al., 2004; Telles et al., 2019). Our series shows a singular etiologic homogeneity, with the exception of an American study (Townsend et al., 2015). It is interesting to note that, although tuberculosis is a frequent opportunistic infection in French Guiana, and it is identified as a cause of HLH in other series, here it was not found to be associated with HLH. Although HLH in HIV patients are rare, systematic probabilistic first line antifungal treatment with liposomal amphotericin B seems justified given our results and those of the literature (De Lavaissière et al., 2009; Castelli et al., 2015; Subedee and Van Sickels, 2015; Townsend et al., 2015). A bibliographic review of HLH secondary to histoplasmosis in HIV-infected patients reports 34 cases with a mean age of 39 ± 11 years (Table 3). The immunosuppression was severe with a median CD4 count of 16 (IQR: 7–39)/mm^3^. Cases were defined on the basis of 4 to 8 HLH-2004 diagnostic criteria. Systemic antifungal treatment was mentioned in 18 of 34 patients. The mortality rate was 14 of 34 patients (41%).

**Table 3:** Case reports and case series on reactive hemophagocytosis due to histoplasmosis in HIV patients.

**References**	**Publication year**	**Country**	**Number of cases**	**Sex**	**Age**	**Number of HLH criteria**	**CD4 count**	**Treatment of HLH**	Outcome^*^
Majluf-Cruz et al. (1993)	1993	Mexico	3	NA	NA	NA	NA	Amphotericin B Fluconazole	1/3 died
Koduri et al. (1995)	1995	USA	6	NA	NA	5	44	Immunoglobulins	3/6 died
Chemlal et al. (1997)	1997	France	1	M	50	4	34	NA	NA
Kumar et al. (2000)	2000	India	1	M	40	4	39	NA	Died
Gil-Brusola et al. (2007)	2007	Spain	1	M	33	4	66	Levofloxacine+Imipenem	Survived
Guiot et al. (2007)	2007	Puerto-Rico	1	M	43	4	39	Liposomal amphotericin B + itraconazole	
Sanchez et al. (2007)	2007	USA	1	M	61	5	4	Liposomal amphotericin B + itraconazole + Anti-TB	Survived
De Lavaissière et al. (2009)	2009	France	1	M	33	6	13	Liposomal amphotericin B + itraconazole	Survived
Vaid and Patel (2011)	2011	UK	1	M	27	5	153	Antifungal treatment	Died
Chandra et al. (2012)	2012	India	1	F	38	4	NA	Ketoconazole	Survived
Telfer and Gulati (2012)	2012	USA	1	M	28	5	12	Voriconazole	Died
Huang (2014)	2014	USA	1	M	25	5	4	Dexamethasone+antifungal treatment	Survived
Townsend et al. (2015)	2015	USA (7) Mexico (1) El Salvador (1)	9	7M+2F	43.9	5	NA	Liposomal amphotericin B + itraconazole +prednisone	7/9 died
Castelli et al. (2015)	2015	USA	1	M	32	8	3	Liposomal amphotericin B + itraconazole	Survived
Subedee and Van Sickels (2015)	2015	USA	1	F	42	7	40	Liposomal amphotericin B + itraconazole	Survived
Nieto et al. (2016)	2016	Colombia	1	M	33	NA	NA	Amphotericin B + steroids	Survived
Gómez-Espejo et al. (2017)	2017	Venezuela	1	M	23	6	7	Liposomal amphotericin B + immunoglobulins +prednisone	Survived
Ocon et al. (2017)	2017	Guyana/USA	1	M	49	6	7	Liposomal amphotericin B + itraconazole+ IL-1 antagonist+dexamethazone	Survived
Loganantharaj et al. (2018)	2017	Dominican Republic	1	M	46	NA	NA	Liposomal amphotericin B	Survived
Asanad et al. (2018)	2018	El Salvador/USA	1	M	48	7	20	Dexamethazone+Liposomal amphotericin B	Survived
Tsuboi et al. (2019)	2019	Venezuela/Japan	1	F	56	6	13	Liposomal amphotericin B + itraconazole+antiretroviral therapy	Survived

* *In total, 41% (14/34) died*.

In contrast with other reports, specific treatment of HLH were rarely used in our series, but in most cases the evolution was favorable (Silva and França, 2014; Townsend et al., 2015; Le Joncour et al., 2016; Usman et al., 2016). This difference is probably linked to the fact that whereas most specific treatments of HLH are used for non-infectious triggers of an autoimmune nature, in our series the triggers were infectious.

Our series presents severe forms of HLH with 5 cases requiring ICU and, in these, a mortality below 50%. The mortality results of our series (14% globally and 17% only for disseminated histoplasmosis cases) seems lower than the mean mortality rates of HLH in HIV patients with histoplasmosis, evaluated at 41% for HLH linked to disseminated histoplasmosis (Koduri et al., 1995; Chemlal et al., 1997; Kumar et al., 2000; Gil-Brusola et al., 2007; Guiot et al., 2007; De Lavaissière et al., 2009; Vaid and Patel, 2011; Chandra et al., 2012; Huang, 2014; Castelli et al., 2015; Subedee and Van Sickels, 2015; Townsend et al., 2015; Le Joncour et al., 2016; Usman et al., 2016). The hypothetical explanation would be that knowledge of the local epidemiology leads to the use of an aggressive diagnostic and therapeutic strategy with a rapid first line treatment strategy using empirical systemic antifungals targeting disseminated histoplasmosis, the most frequent opportunistic infection in HIV patients in French Guiana. Perhaps the rapid response to Liposomal amphotericin B explains this lower case-fatality in comparison to HLH cases linked to Lymphoma or Kaposi. Finally, although the cascade of care has greatly improved, it is noteworthy that during the study period 12 of the 57 reported cases of histoplasmosis in French Guiana (Nacher et al., 2020a,b) were HLH, which emphasizes that the problem is not an anecdotal one if 1 in 5 patients with disseminated histoplasmosis are concerned. The opportunistic pathogen ecology in French Guiana is similar in the Guianas and the Amazon, and for histoplasmosis the high incidence observed in French Guiana can also be observed in much of South and Central America (Adenis et al., 2018). The present results therefore suggest that in HIV-infected patients with HLH in these regions should benefit from active search for *H. capsulatum* and from prompt antifungal treatment with amphotericin B, preferably in its liposomal formulation.

## Conclusion

HLH is usually considered to be rare and usually occurs in severely immunocompromised patients. The diagnosis is difficult and therefore presumably underestimated. The present results suggest that it is indeed more frequent than previously thought. The more frequent use of the HScore, in addition to HLH-2004 criteria, seems pertinent given the actual state of knowledge.

For the first time, in the context of French Guiana, disseminated histoplasmosis appears as the most frequent etiology of HLH in HIV patients. Hence the rapid initiation of probabilistic first line antifungal therapy with liposomal amphotericin B seems pertinent and beneficial on the vital prognosis of HIV patients with HLH. This further emphasizes the importance of early diagnosis and treatment of disseminated histoplasmosis. It also suggests that the incidence of disseminated histoplasmosis-associated HLH in patients with advanced HIV in Latin America is presumably far more frequent than the literature suggests. The recent reports from Latin American countries are only the tip of the problem and greater awareness will presumably unveil the true magnitude of the problem (Adenis et al., 2018; Caceres et al., 2019).

## Data Availability Statement

The raw data supporting the conclusions of this article will be made available by the authors, without undue reservation, to any qualified researcher.

## Ethics Statement

All HIV-infected patients followed at Cayenne Hospital are included in the DAT'AIDS database. This database allows the clinical care of HIV patients with the eNADIS computerized medical records. It is part of the French Hospital Database on HIV, a national cohort which has led to numerous scientific publications. This database received approval from the Commission Nationale Informatique et Libertés (CNIL) since 27/11/1991. All included patients signed an informed consent form after presentation of the database by the treating physician.

## Author Contributions

DN and AA: conception. DN, MN, LE, AM, MD, DB, RB, KD, PA, FD, PC, and AA: data collection and study conduct and manuscript validation. DN, MN, and AA: manuscript drafting. MN: final manuscript validation. All authors contributed to the article and approved the submitted version.

## Conflict of Interest

The authors declare that the research was conducted in the absence of any commercial or financial relationships that could be construed as a potential conflict of interest.
